# P-1428. Risk Factors for Bacterial Infections After Firearm Injury

**DOI:** 10.1093/ofid/ofae631.1603

**Published:** 2025-01-29

**Authors:** Jerry Yang, Chih Chun Tung, Melike Harfouche, Anthony Harris, Jonathan Baghdadi

**Affiliations:** University of Maryland Medical System, Baltimore, Maryland; University of Maryland School of Pharmacy, Baltimore, Maryland; University of Maryland School of Medicine, Baltimore, Maryland; University of Maryland School of Medicine, Baltimore, Maryland; University of Maryland School of Medicine, Baltimore, Maryland

## Abstract

**Background:**

Every day, over 300 Americans sustain firearm injuries, but prevalence estimates for bacterial infection after these injuries vary widely.

**Figure:**
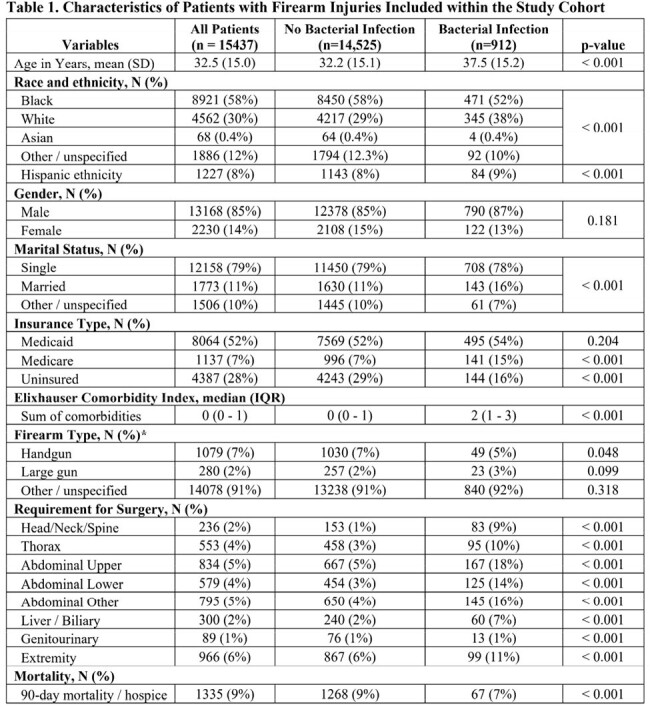

**Methods:**

We studied a retrospective cohort of adults with firearm injuries using administrative data from 233 US hospitals in the Premier database from 1/2019 through 4/2021. Encounters for firearm injury were identified by ICD-10 diagnosis codes. The index encounter for firearm injury and subsequent encounters at the same hospital within 90 days were assessed for bacterial infection, defined by a positive clinical culture and at least 4 days of antibiotic therapy or antibiotics until death. Risk factors, including firearm type, intent of injury, and injury location were identified using diagnosis and procedure codes. Relationships between risk factors and infection were evaluated with multivariable logistic regression.

**Figure:**
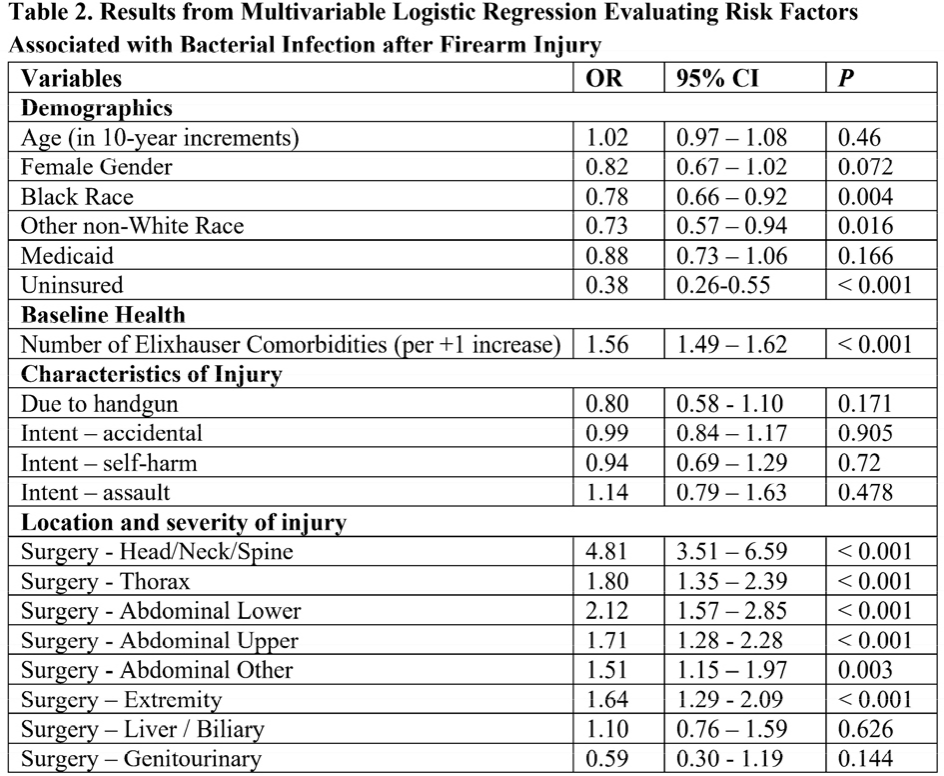

**Results:**

15,437 patients with a medical encounter for firearm injury were identified, including 2,610 (17%) who required surgery during the index encounter and 912 (6%) who developed bacterial infection. 14% of bacterial infections (125/912) developed during 90-day follow-up after the index encounter (median time to presentation 20 days, IQR 8 – 45), including 1% of patients who had surgery during the index encounter (37/2610). On average, patients who developed bacterial infection were 32.5 years old (sd 15.0), 87% men (790/912), 78% single marital status (708/912), and 54% had unintentional firearm injuries (490/912). In multivariable analysis, injury requiring head, neck, or spine surgery (OR 4.8, 95% CI 3.5 – 6.6), lower abdominal surgery (OR 2.1, 95% CI 1.6 – 2.8), or thoracic surgery (OR 1.8 (95% CI 1.4 – 2.4) were the strongest risk factors for bacterial infection. Mortality was 8.7% (1335/15437), including 7.4% (67/912) of patients with bacterial infections. In multivariable analysis, bacterial infection was not significantly associated mortality (OR 0.8, 95% CI 0.6 - 1.1).

**Conclusion:**

Bacterial infection is an infrequent complication of firearm injury that tends to present during initial hospitalization. Patients undergoing head, neck, spine, large bowel, or chest surgery are at highest risk overall, though new postoperative infection is rare at follow-up.

**Disclosures:**

**Anthony Harris, MD, MPH**, Innoviva: Advisor/Consultant|UpToDate: Infection Control Editor

